# Small Packages, Big Returns: Uncovering the Venom Diversity of Small Invertebrate Conoidean Snails

**DOI:** 10.1093/icb/icw063

**Published:** 2016-07-01

**Authors:** J. Gorson, M. Holford

**Affiliations:** 1 *Department of Chemistry, Hunter College, The City University of New York, Belfer Research Building, NY, 10021 USA; † Departments of Biology, Chemistry, and Biochemistry, The Graduate City, The City University of New York, NY, 10016 USA; ‡ Invertebrate Zoology, Sackler Institute of Comparative Genomics, American Museum of Natural History, NY, 10024 USA

## Abstract

Venomous organisms used in research were historically chosen based on size and availability. This opportunity-driven strategy created a species bias in which snakes, scorpions, and spiders became the primary subjects of venom research. Increasing technological advancements have enabled interdisciplinary studies using genomics, transcriptomics, and proteomics to expand venom investigation to animals that produce small amounts of venom or lack traditional venom producing organs. One group of non-traditional venomous organisms that have benefitted from the rise of -omic technologies is the Conoideans. The Conoidean superfamily of venomous marine snails includes, the Terebridae, Turridae *(s.l),* and Conidae. Conoidea venom is used for both predation and defense, and therefore under strong selection pressures. The need for conoidean venom peptides to be potent and specific to their molecular targets has made them important tools for investigating cellular physiology and bioactive compounds that are beneficial to improving human health. A convincing case for the potential of Conoidean venom is made with the first commercially available conoidean venom peptide drug Ziconotide (Prialt®), an analgesic derived from *Conus magus* venom that is used to treat chronic pain in HIV and cancer patients. Investigation of conoidean venom using -omics technology provides significant insights into predator-driven diversification in biodiversity and identifies novel compounds for manipulating cellular communication, especially as it pertains to disease and disorders.

## Introduction

**Fig. 1.**
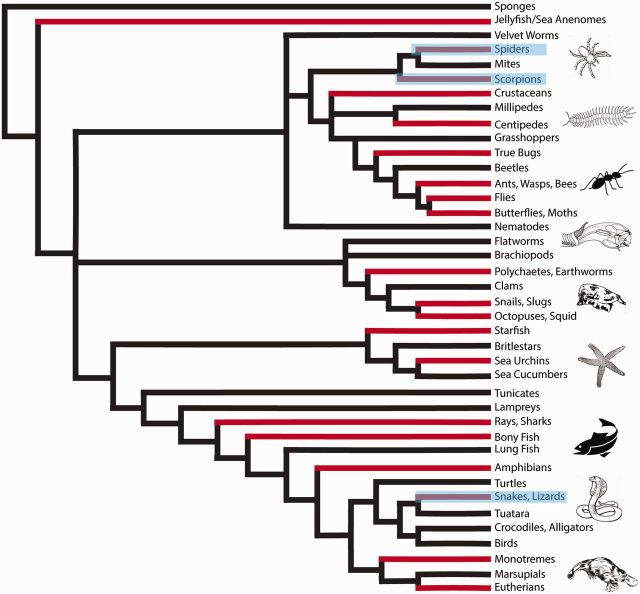
Biodiversity of venomous taxa. Phylogenetic reconstruction of the tree of life highlighting venomous organisms. Grey bars represent the clades that include venomous organisms. Highlighted clades represent the traditionally studied venomous taxa (scorpions, spiders, snakes, and lizards).

Venom is defined as any exogenous substance that is used to elicit an adverse effect in its target, and as a result a wide range of organisms from notorious snakes to lesser known leeches and bees are considered venomous (Fig. 1; Escoubas and King 2009; Casewell et al. 2013; King 2015; Petras et al. 2015). Historically, organisms used in venom research were chosen opportunistically, based on size and ease of collection, which largely focused on vertebrates, specifically snakes. Two genera of snakes account for almost 40% of all published venom toxin sequences in elapid snake venom research (Fry et al. 2008). Remarkably, one easy to collect genus (*Naja*) has been used to identify 40% of all three-finger snake venom toxins (3FTxs) sequenced. Only three studies have used harder to milk and less studied, non-front-fanged snakes to investigate 3FTx bioactivity (Fry et al. 2003; Pawlak et al. 2006, 2009; King 2015). The venom research strategy of size and accessibility can neglect the ecology, morphology, or evolutionary relatedness between organisms, resulting in a diversity of venomous animals, such as invertebrates, being effectively ignored (Modica and Holford 2010; Puillandre and Holford 2010; von Reumont et al. 2014b).

Invertebrates are underrepresented in venom research. Spiders, which are the most diverse group of venomous animals, with about 45,000 species, make up less than 5% of all venom research studies (Nentwig 2013). Analogous to the within taxa bias seen in snakes, of the 17,000 species of scorpions described, only ∼50 species have had their venom investigated (King 2015). It can be argued that the venom research bias existed largely due to lack of technological methods for effectively collecting and characterizing small quantities of venom. The deficiency of invertebrates has led to a dearth of information that has hindered venom research. However, recent technological advancements in the field of molecular biology and proteomics has increased the representation of marine cone snails, sea anemones, bees, and ants in venom studies (Norton and Olivera 2006; Moran et al. 2008; Barlow et al. 2009; Casewell et al. 2013; Sanggaard et al. 2014; Zhang et al. 2015). Extensive research on a broad range of organisms is imperative in order to effectively derive and test hypotheses about venom as it relates to species diversification, predator–prey interactions, and to describe the immense biodiversity of animals found on Earth (Fig. 1).

## Rise of -omics

**Fig. 2.**
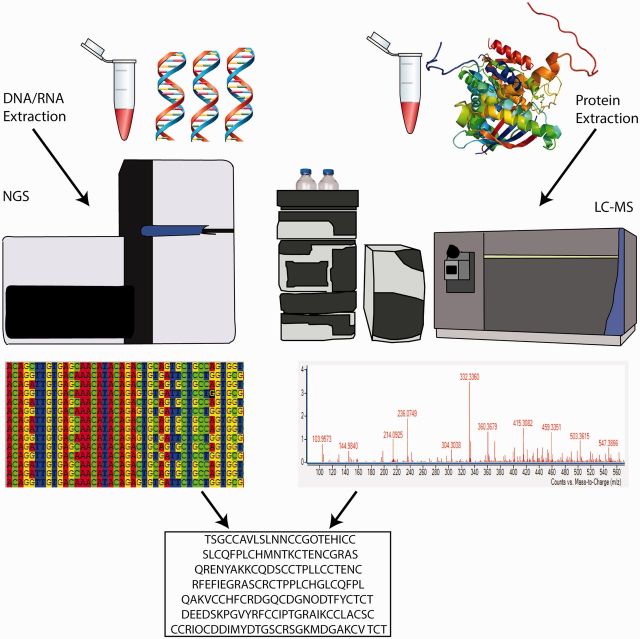
Venomics: an integrated NGS and proteomic strategy. An integrated multi -omics approach using genomic, transcriptomic, bioinformatic, and proteomic protocols to identify venom proteins and peptides. Application of a combined -omics strategy validates *de novo* venom peptide/protein identification and provides robust data to test hypotheses related to venom evolution and ecology. The sequences shown at the bottom are an example of a validated peptide database obtained from NGS and proteomics.

Early research on venom relied heavily on identifying proteins using Edman degradation and mass spectrometry (MS; Perkins et al. 1993a, 1993b). In conjunction with fractionation, MS allowed for the separation and identification of individual venom components. Development of soft ionization methods in the late 1980s, such as electrospray ionization (ESI; Fenn 2003), and matrix-assisted laser desorption (Karas and Hillenkamp 1988; Tanaka 2003) have revolutionized biological research. In particular, the ability to identify proteins directly from MS data is a powerful capability that soon demonstrated to be crucial for analyzing venoms. Quickly thereafter, research groups started to implement MS techniques to characterize snake venom (Perkins et al. 1993a, 1993b). MS approaches also enable the identification of isoforms of venom peptides, slight variations in sequences, and post translational modifications (Craig et al. 1999; Escoubas et al. 2008; Safavi-Hemami et al. 2014; Petras et al. 2015). Recent advancements in MS protocols have produced what are referred to as top-down methods, in which whole intact venom components can be identified (Breuker et al. 2008; Ueberheide et al. 2009; Anand et al. 2014; Sunagar et al. 2016). Although groundbreaking, in some organisms a proteome-only MS approach can be problematic. MS requires extraction of venom, which is impractical for organisms that do not readily store venom for delivery and other organisms that have hard-to-access venom delivery systems that prevent stimulation or extraction (von Reumont et al. 2014a). Additionally, MS methods for determining primary venom peptide sequences are largely dependent on downstream data analyses of source databases, such as Mascot or Genbank (Perkins et al. 1999; Bhatia et al. 2012). For model organisms with a rich complement of sequence databases, such as humans, mice, or drosophila, this is not an issue. In the case of non-model organisms, the application of MS methods for primary sequencing is severely limited by the database used. Non-model systems generally require *de novo* sequence assembly and source databases that are either missing or deficient. As a result, an integrated strategy, termed venomics (Calvete et al. 2007; Calvete 2014; Eichberg et al. 2015), in which MS proteomics is combined with next generation transcriptomic or genomic sequencing and bioinformatic methods is necessary to validate characterization of *de novo* venom peptides found in non-model organisms and to paint the full canvas of venom evolution and variation (Fig. 2; Fry et al. 2013; Sunagar et al. 2016). Using the multi-omic integrated venomic strategy, venom research has become more accessible to smaller, harder to collect, and understudied venomous taxa. The integrated venomic strategy has also broadened the scientific community engaged in venom research from traditional chemists and pharmacologists looking for bioactive compounds for drug discovery and development, to evolutionary biologists looking for anatomical and molecular characters to understand venom evolution through various taxa over time (Duda and Palumbi 1999; Moran et al. 2008; Favreau and Stöcklin 2009; Elmer et al. 2010; Koh and Kini 2012; Otvos et al. 2013; Gorson et al. 2015; Jouiaei et al. 2015; Zhang et al. 2015).

The honey bee, *Apis mellifera*, was the first venomous organism to have a fully sequenced genome using Sanger sequencing (Weinstock et al. 2006). Since then, the development of next generation sequencing (NGS) high throughput techniques has allowed rapid sequencing of other venomous organisms. The genomes of tarantula *Acanthoscurria geniculate* (Sanggaard et al. 2014), scorpion *Mesobuthus martensii* (Cao et al. 2013), velvet spider *Stegodyphus mimosarum* (Sanggaard et al. 2014), fire ant *Soenopsis invicta* (Wurm et al. 2011), and king cobra *Ophiophagus hannah* (Vonk et al. 2013) have all been sequenced using NGS technologies. With multiple platforms available, such as Illumina (Illumina, Inc., San Diego, California), 454 (Roche Applied Science, Penzberg, Germany), SOLiD (ThermoFisher Scientific, Waltham, Massachusetts), and Ion Torrent (ThermoFisher Scientific, Waltham, Massachusetts), genome sequencing of venomous organisms is becoming both accessible and affordable. However, genomics alone does not provide enough information for determining the exact mode and tempo of gene expression and does not give significant insight into differential gene expression within various tissue types (Sunagar et al. 2016).

While genomics is the study of the complete DNA composition of an organism, venom gland transcriptomics is the sequencing of mRNA specific to the venom gland or secretory tissue of a venomous organism and therefore a glimpse at the specific venom cocktail being used at the time by the animal (Durban et al. 2011; Dutertre et al. 2014; Gorson et al. 2015; Sunagar et al. 2016). Both transcriptomics and genomics enable the identification of certain domains of a venom protein, such as the signal and pre-pro regions that are rarely identified on the proteomic level as they are cleaved off after translation (Duda and Palumbi 1999; Espiritu et al. 2001; Kaas et al. 2010; Robinson and Norton 2014; Sunagar et al. 2016). Employing an integrated venomic strategy has enabled researchers to resolve previously unanswerable questions, such as identifying a correlation between varying venom compositions and differences in ecological and environmental factors. Several studies employing a combined genomics and transcriptomics approach have looked at venom variation between different developmental stages in snakes (Durban et al. 2011; Durban et al. 2013; Gibbs et al. 2013). Specifically, proteomics and transcriptomics were used to show venom variation in various populations of the Southern Pacific Rattlesnake, *Crotalus oreganus helleri* in the United States (Sunagar et al. 2014). Lectin β-chains, which are generally undergoing positive selection, were found to be evolving under negative selection in the *C. oreganus helleri* rattlesnake population found on Catalina Island (in the Pacific Ocean; Sunagar et al. 2014). The integrated venomics approach used in this study revealed that there can be different evolutionary selection pressures acting on different venom classes depending on the population site. In another study that used an integrative venomics approach, it was found that there were significant differences in the mature peptides being produced in different samples of venom from *Conus consors* (Biass et al. 2015). Proteomics and transcriptomics were used to analyze *C. consors* venom at three different stages: venom milked from the snail, venom extracted from the venom gland, and venom expressed in the transcriptome, effectively tracing the venom production and delivery process from the venom glad tissue to the point of venom envenomation of the prey. The surprising result was that the cocktail of venom peptides identified in the transcriptome, in the venom produced within the venom gland, and in the venom injected into the prey were not heavily correlated. Each venom compartment was distinctive in terms of peptide and protein content. This study emphasizes the complexity of the venom mechanism of Conodiean snails and indicates what is being secreted in the venom is not necessarily the same as what is being produced in the gland (Biass et al. 2015).

Advances in -omic technologies have increased the breadth of research being done on all organisms and have advanced research of non-model organisms.

## Characterizing Conoidean Venom Evolution and Variation

The technological -omics advancements removed the barrier requiring large amounts of crude venom extracts and smaller-sized taxa such as centipedes, certain sea anemones, ants, and small scorpions have become the focal point of an ever increasing number of venom studies (Putnam et al. 2007; Moran et al. 2008; Calvete et al. 2009; Cao et al. 2013; Sanggaard et al. 2014; Xu et al. 2014). One non-model organism that has received a lot of attention using venomic technologies is the venomous marine snails in the Conoidean superfamily.

**Fig. 3.**
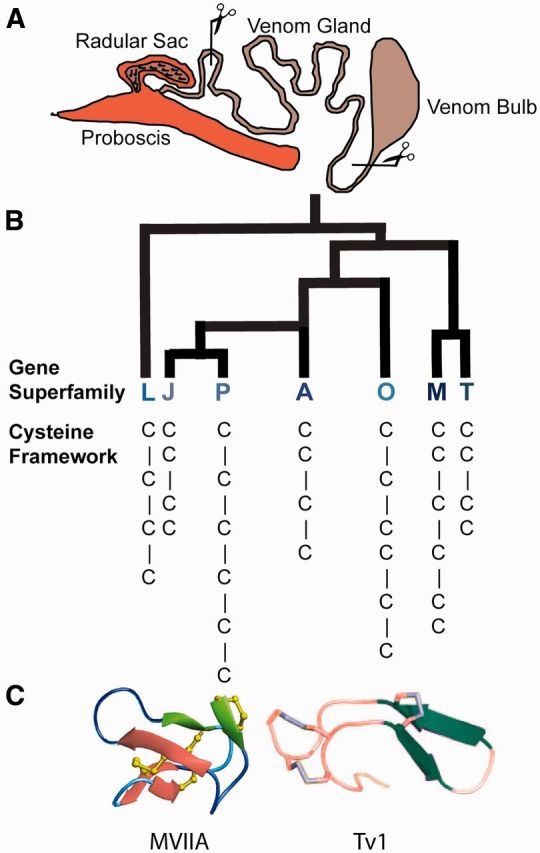
Conoidean venom characterization. (A) A generic representation of the Conoidean venom apparatus, which includes: a venom bulb that is contracted to push the venom through the venom gland, where the venom is being produced, a radular sac that contains hollowed teeth (harpoons) that are used to inject venom into the prey, and a proboscis that extends several times beyond the snails body size to deliver venom-filled radula to a prey target. Scissors shown represent the dissection of the venom duct for downstream analysis by transcriptomic, genomic or proteomic methods. (B) Identification of *Conus* venom peptide superfamilies and cysteine frameworks. (C) Conoidean venom peptides selected for bioactivity characterization. MVIIA is a peptide from *Conus magus* venom that produced the ziconotide (Prialt®) drug that is commercially available. MVIIA is in the O1 conotoxin gene superfamily and has a VI/VII cysteine framework. Tv1 is a peptide from *Terebra variagata* that has a cysteine pattern similar to the M-superfamily in cone snails and has a III cysteine framework, but has a peptide fold of antiparallel beta hairpins that is unique to known venom peptides. Tv1 and MVIIA are very distinct peptides illustrating the disparate complexity of Conoidean venom peptides.

Conoidean snails are slow moving predators and therefore rely heavily on the efficacy of their venom (Azam et al. 2005; Yao et al. 2008; Kendel et al. 2013; King 2015). The dependence on venom for prey capture has led conoidean venom peptides to achieve incredible molecular specificity (Olivera 2002; Olivera et al. 2014). Conoideans subdue their prey using a venom apparatus made up of a proboscis, radular tooth, a radular sac, venom gland, and venom bulb (Fig. 3(A); Taylor 1990; Kantor et al. 2000; Modica and Holford 2010; Kantor and Puillandre 2012). Cone snails (*Conus*) are the most studied in the Conoidea (Puillandre et al. 2014; Puillandre et al. 2015); however, *Conus* comprises only ∼5% of the biodiverse group of venomous marine snails (Olivera et al. 1999; Holford et al. 2009; King 2015). Other non-*Conus* Conoideans, such as the Turridae (*s.l.*) family, which has more recently been divided into seven family groups (Tucker and Tenorio 2009; Bouchet et al. 2011), and the Terebridae family, also produce venom (Heralde et al. 2008; Aguilar et al. 2009; Gonzales and Saloma 2014; Gorson et al. 2015; Moon et al. 2016). Cone snails and terebrids dwell in shallow-water tropical marine habitats, while the majority of turrids can be found at greater depths (>200m; Taylor 1977). Terebrids and turrids (some less than 3 mm in length) have incredibly small venom ducts, producing limited amounts of venom, which initially inhibited their characterization. Using an integrated venomics strategy, venom research of terebrids and turrids has become more feasible (Castelin et al. 2012; Kendel et al. 2013; Gonzales and Saloma 2014; Gorson et al. 2015; Moon et al. 2016).

Conoidean venoms are a complex mixture of small molecules, peptides and proteins (Norton and Olivera 2006; Gonzales and Saloma 2014; Gorson et al. 2015; Neves et al. 2015). Each Conoidean venom consists of >100 different peptides which contain a signal sequence, followed by a pro region, and a mature peptide at the C-terminus (Olivera et al. 1999; Lavergne et al. 2015). As cone snails are well studied, 3000 different peptides (conotoxins) have been identified from *Conus* venoms since the 1970s (Conoserver.org). The majority of conotoxins have been classified into venom gene superfamilies by examining the sequence identity of the signal sequence (Jacob and McDougal 2010; Robinson et al. 2014; Robinson and Norton 2014). A similar process is being used to characterize turrid and terebrid venom peptide superfamilies (Fig. 3(B); Heralde et al. 2008; Gonzales and Saloma 2014; Gorson et al. 2015). Different venom peptide superfamilies generally have distinct physiological targets and high specificity for those targets (Olivera et al. 1999). While there are similarities between the venoms of *Conus*, inter- and intraspecific variation exists, such as differences in the proportions of cysteine frameworks or venom gene superfamilies (Duda and Palumbi 1999; Olivera et al. 1999; Jakubowski et al. 2005; Romeo et al. 2008; Abdel-Rahman et al. 2011). Due to the high rates of non-synonymous mutations and the early divergence of Conoidean families, the venoms of terebrids and turrids (*s.l.*) vary significantly from the venoms of cone snails (Powell 1966; Duda and Palumbi 1999; Puillandre and Holford 2010; Gorson et al. 2015). The disparity in *Conus* and terebrid venom was recently revealed by looking at the variation in venom peptide superfamilies between *Triplostephanus anilis* and *Terebra subulata* (Gorson et al. 2015). Fourteen terebrid venom gene superfamilies were identified in the two terebrid species (TA, TB, TC, TD, TE, TF, TG, TH, TI, TJ, TK, TL, and TM). Of the fourteen terebrid superfamilies described, only one, TM, is homologous to a superfamily found in *Conus marmoreus* (H superfamily*;*Robinson and Norton 2014). The divergence of *Conus* and terebrid venom gene superfamilies suggests that terebrid venom peptides will have different structural features and physiological targets from *Conus*, thereby increasing the pool of bioactive compounds that can be explored for discovery of novel therapeutic drugs. Conoidean venoms are exceedingly effective candidates for drug discovery as they are: (1) rapid acting, (2) highly selective, and (3) very potent (Fig. 3(C)).

## Potential of Conoidean Venom to Increase Drug Discovery and Development

Many bioactive peptides have evolved as a means of predation or defense, especially in venomous animals (Olivera et al. 1985; Gray et al. 1988; Olivera et al. 1999; Dutertre et al. 2014). The wide variety of biologically active venom peptides are a promising resource for drug discovery (Lewis and Garcia 2003; Favreau and Stöcklin 2009; Twede et al. 2009; Koh and Kini 2012; King 2015; Ortiz et al. 2015). The constant selective pressures acting on venom, due to the effects of the predator–prey arms race (Van Valen 1973; Dawkins and Krebs 1979; Casewell et al. 2013; Holding et al. 2016), enabled venom peptides to develop features to increase stability and prey molecular target affinity. Venom peptides tend to interfere with transmissions of ions in and out of cells, suggesting they would be effective tools for manipulating ion channel driven cell disorders such as pain or cancer (Miljanich 2004; Vetter and Lewis 2012; Lang et al. 2014). These properties make venom peptides more appealing than artificially or chemically conceived peptide-like compounds for which bioactivity is not guaranteed (Uhlig et al. 2014).

The past two and a half decades have seen an increase in the number of projects that are taking advantage of the Earth’s amazingly biodiverse group of venomous organisms to develop novel drugs, to create tools for diagnosing human diseases, and to create probes to help advance the study of molecular receptors and physiological pathways (Nisani et al. 2007; King 2011; Diochot et al. 2012; Casewell et al. 2013). As reptiles were the most accessible venomous organisms for quite some time, the majority of approved venom drugs were discovered from snake venom. Specifically, snake venom proteins targeting thrombin, integrin, and fibrinogen receptors were discovered (King 2011; Koh and Kini 2012). Captopril®, an angiotensin-converting enzyme (ACE) inhibitor synthesized to mimic a venom peptide from Brazilian lancehead snakes, is a breakthrough drug that validates venom-based drug discovery research (Cushman and Ondetti 1991). The venomic strategy has made it more affordable and practical to examine non-model venomous organisms for peptide or protein components that can lead to new therapeutics. Investigating more venomous organisms will greatly increase the amount of compounds available for drug discovery and development (Puillandre and Holford 2010; Casewell et al. 2013; King 2015).

**Fig. 4.**
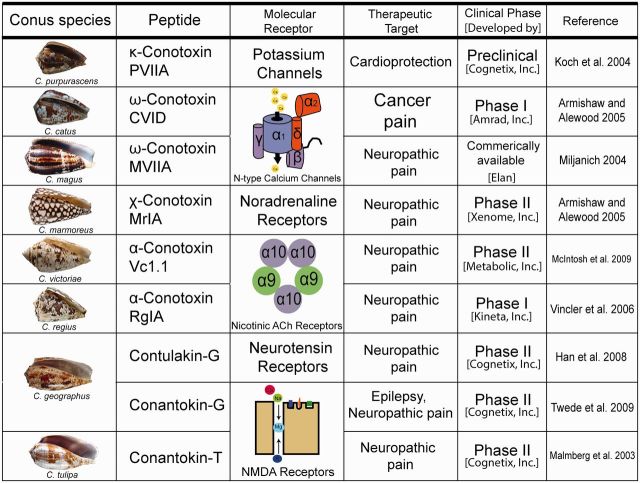
Snail venom peptides to drugs. Conoidean venom peptides under drug development.

The >1 million estimated venom peptides expressed in conoidean venom are an immense resource for discovering novel compounds for therapeutic drug development. The majority of conoidean venom peptides are disulfide-rich molecules that have been shown to manipulate voltage and ligand gated potassium (K^+^), calcium (Ca^2+^), and sodium (Na^+^) channels, as well as nicotinic acetylcholine receptors, and noradrenaline transporters (Olivera 2002; Terlau and Olivera 2004; Becker and Terlau 2008; Fig. 4). As mentioned previously, ion channels and receptors are the molecular targets for cancer, pain, and other debilitating human disorders (Wood et al. 2004; Veiseh et al. 2007; Gkika and Prevarskaya 2011; Dave and Lahiry 2012; Lang et al. 2014). Ziconotide (Prialt®), a peptide from the venom of *Conus magus*, was approved for commercial use by the Food and Drug Administration in 2004 and is the first non-opiod analgesic (Olivera 2000; Miljanich 2004; Schmidtko et al. 2010). Similar to MVIIA, CVID, MrIA, Vc1.1, RgIA, Contulakin-G, and Conantokin-T are peptides synthesized from the natural secretions of *Conus* that are currently undergoing clinical trials to determine of their potential as pain therapeutics (Fig. 4; Malmberg et al. 2003; Miljanich 2004; Armishaw and Alewood 2005; Vincler et al. 2006; Han et al. 2008; McIntosh et al. 2009). Although most *Conus* peptides in pharmaceutical development are being used as analgesics, PVIIA shows promise as a therapy for myocardial infarction, and Conantokin-G for epilepsy (Fig. 4; Koch et al. 2004; Armishaw and Alewood 2005; Twede et al. 2009).

## Conclusion

The progress of -omics technologies triggered a domino effect in venom research. An integrated venomics strategy has enabled a broader range of venomous organisms, many of which are non-model organisms, difficult to acquire, and contain limited amounts of venom, to be examined and ultimately contribute to the understanding of venom evolution in biodiversity. Without the vast technological molecular improvements in the last decade, studies would most likely still revolve around snakes, spiders, and scorpions. The increasing amount of venom peptides identified from Conoideans that are now in clinical trials, demonstrates the importance of expanding the diversity of venomous species examined (Fig. 4). As -omics technology continues to improve, it will be easier and cheaper to add more species to the pool of venomous organisms under investigation, enabling researchers to resolve questions about venom convergence across the animal kingdom and increase the quantity of peptides available for drug discovery and development for the benefit of human health.
